# The evolutionary traverse: a causal analysis

**DOI:** 10.1007/s00191-016-0483-3

**Published:** 2016-11-10

**Authors:** David Haas

**Affiliations:** Graz Schumpeter Centre, University of Graz, Universitätsstrasse 15/FE, Graz, A-8010 Austria

**Keywords:** Technical change, Diffusion, Competitive selection, Adaptive growth and employment, Resource constraint, B15, B25, E1, O11, O33

## Abstract

This paper explores the process of adaptation to new methods in a simple model where the growth rate of labour supply is exogenously given and constant. It shows that competition for a primary input in short supply changes the mechanism of adaptation and its consequences: If surplus labour exists, differential capacity accumulation effectuates adaptation and leads to a logistic replacement pattern; but if labour is in short supply, ‘growth predation’ undermines the former mechanism and leads to an exponential replacement pattern. The consequences of the quantitative adjustment mechanisms for aggregate growth are discussed by means of a ‘causal analysis’, which focuses on the properties of the traverse between two full-employment steady states. The analysis reveals that different types of new methods lead to different adaptation paths and results. Overall, adaptation entails unsteady growth and it is not always the case that the diffusion of a new method boosts aggregate growth.

## Introduction

We study the evolutionary traverse in a one-commodity model where labour is supplied inelastically. The aim of this exercise is to clarify the consequences of a resource constraint for the evolutionary adjustments triggered by the arrival of new methods of production.

In the evolutionary approach to technical change adaptation is recognized as a selection process, where differences in the efficiency of used methods, certain routines of firms and the economic as well as the institutional environment determine the rise of superior methods and the decline of inferior ones. This ‘restructuring’ is seen as a vital source of growth and technical change and typically exhibits a logistic pattern (Metcalfe 1998, 2008).

In simple variation-cum-selection models which prefer a macroeconomic interpretation, such as Nelson and Winter (1982, chap. 10) and Silverberg (1984), labour supply conditions play an important role for the selection process and its effects. For example, Englmann (1992) shows that the response of the real wage rate to selection dynamics shapes the overall employment effect of the diffusion of a new method.

Although different wage adjustment mechanisms are explored, the common assumption made in simple variation-cum-selection models is that there always is enough labour to fully employ the capital stock. In this context, Metcalfe and Steedman (2013) argue that this implicit assumption of surplus labour is not as innocuous as it may seem. They draw attention to the fact that the nature of adaptation depends on whether surplus labour exists or not: If the supply of labour is unlimited, adaptation is driven by the differential abilities to invest by means of retained profits. But if the supply of labour is fixed and in short supply, a different mechanism called ‘growth predation’ here effectuates adaptation. It relies on the idea that firms using the superior method can attract workers employed elsewhere through offering wages that are higher than what firms employing inferior methods can pay. Through this the superior method displaces the inferior one not only in relative but also in absolute terms through depriving the inferior one of the basis of its very existence. A further important difference between the two cases is that in the surplus labour case the amount and distribution of capital limits total output, whereas in the labour shortage case the amount and distribution of labour limits total output. This has important implications for the growth effect of competitive selection, which depends both on the bias of the innovation and the state of labour supply; see Haas (2015) for a typology of new methods and their growth effect for the case of surplus labour.

The paper develops the idea of ‘growth predation’ in a simple model in order to put into sharp relief the effects of a resource constraint that potentially limits output at the population level. A causal analysis helps to shed light on the crucial forces and features of what may be called an ‘evolutionary traverse’, i.e. the path from one full-employment steady state to another when the original steady state is disturbed by an innovation. With minor differences, this approach resembles Schumpeter’s (1934) analytical scheme for understanding economic development based on the concept of what he called the circular flow (Kurz 2008).

In line with Metcalfe and Steedman (2013), adaptation is brought about by output share dynamics only; the problem of imitation is not discussed. In contrast to Metcalfe and Steedman (2013), we assume that capital is not circulating but perennial and non-malleable. This means that capital does not depreciate and cannot be transformed to serve other purposes. Thus in the case of a labour shortage some firms have to accept that part of their equipment lies idle. Note that under-utilisation of capital results from a lack of complementary means to employ it and not from a lack of effective demand; the latter problem is neglected in this paper.

The main findings include: (i) The implementation of certain types of new methods causes unemployment which is relieved through the above-normal growth potential of ‘new’ firms; (ii) as long as unemployment prevails, adaptation through differential accumulation features a logistic replacement pattern, a typical feature of evolutionary models, whereas adaptation through ‘growth predation’ leads to an exponential replacement pattern; (iii) the aggregate growth rate depends both on the question of whether there is full employment or not and the bias of the innovation. Growth is unsteady and the diffusion of an innovation is not always expansionary.

The paper is organized as follows: Section 2 presents a simple growth model with two rival firms. In Section 3 the relation between adaptation and growth for different types of innovations is explored by means of a causal analysis. Section 4 concludes. The Appendix shows that the traverse towards a fully automated system differs fundamentally from other cases.

## A simple evolutionary growth model

This section presents a simple ‘macro’ selection model, where labour supply grows at an exogenous rate and firms use distinct technologies. For analytical convenience we deal with the simplest case of two rival groups of firms, namely users of the ‘old’ technology and users of the ‘new’ technology. For a greater ease of reading, we will call the former group ‘old firm’ (indexed by 1) and the latter one ‘new firm’ (indexed by 2).

As we are concerned with basic relationships in evolutionary selection models and how certain assumptions drive results, we work with a set of premises that is typical for such models. In particular, it is assumed that variety in terms of methods is not renewed through innovation and that there is no imitation.^1^ Rather, economic change at the macro level results exclusively from changes in the economic weight of rival technologies. Through this process of competitive selection the economy as a whole is able to adapt even though there are strong inertial forces at the micro level.^2^

### Production

We assume a closed economy in which firms produce a single good which serves both as an investment and as a consumption good. Output of firm *i* ∈ {1, 2} is determined by the fixed-coefficients method of production

1 $$ x_{i,t} = \min \left\{\frac{L_{i,t}}{l_{i}}; \frac{K_{i,t}}{b_{i}}\right\}, $$

where *x*_*i*, *t*_, *K*_*i*, *t*_ and *L*_*i*, *t*_ are the output, the stock of perennial capital (‘machines’) and employment of firm *i* in period *t*. Because the two rivals use distinct technologies, labour coefficient *l*_*i*_ and *full-utilisation* capital coefficient *b*_*i*_ are firm-specific parameters.

Before production starts, firms hire workers. To avoid the problems of heterogeneous labour and of skill formation, workers are treated as homogeneous in skills and efficiencies and both methods are assumed to require the same type of labour. This is crucial here, because the two rivals compete for the same primary input which is not in unlimited supply. Rather, labour supply *N* grows at a given and constant rate *n* > 0 such that^3^

2 $$ N_{t}=\left( 1+n\right)N_{t-1}. $$

Assume that firm *i* wants to produce full capacity output, which means that its labour demand, or desired employment, is given by

$$L^{d}_{i,t}=\frac{K_{i,t}l_{i}}{b_{i}}. $$

Because the quantity of labour available is limited and inelastic, *total actual* employment, which we denote by $L_{t}= \sum \nolimits _{i}L_{i,t}$, in any case must satisfy the inequality condition

$$L_{t} \le N_{t}. $$

Clearly, if the firm population demands more labour than is available, i.e if $\sum \nolimits _{i}L^{d}_{i,t}> N_{t}$, this condition binds in such a way that at least one firm is rationed on the labour market and hence must produce below full capacity.

How could this be resolved? We follow Metcalfe and Steedman (2013) who argue that an input shortage, faced by the firm population as a whole, may influence different firms differently. The argument is this: Firms which use distinct technologies in general yield different rates of profit; and because of this they differ in their ability to attract workers by offering a wage that is higher than what rivals can pay.

Assume that the ‘new’ firm 2 pays a slightly higher wage rate than firm 1 and as a result is able to satisfy its labour demand. The extent of this wage differential depends on the perfection of the labour market. If workers are fully informed and perfectly mobile, a negligibly small premium will attract enough workers (Metcalfe and Steedman 2013; see also Nelson and Pack, 1999).^4^ For simplicity both nominal wage rates, *w*_1_ and *w*_2_, are rigid and hence stay constant. Because *w*_2_ > *w*_1_, there is an asymmetry in the determinants of firm employment levels given by

3a $$\begin{array}{@{}rcl@{}} L_{1,t} &=& \min \left\{L^{d}_{1,t};N_{t}-L_{2,t} \right\}, \end{array} $$

3b $$\begin{array}{@{}rcl@{}} L_{2,t} &=& \min \left\{L^{d}_{2,t};N_{t}\right\}. \end{array} $$

Equation 3b states that new firm 2 is rationed only if its *own* labour demand exceeds total supply. For the old firm 1 matters are more complex. It is rationed if *total* labour demand exceeds total supply. If this is the case, its employment depends on the accumulated stock of machines of the new firm. This interaction that results from competition for a limited quantity of labour is the basis for the mechanism of ‘growth predation’ (see Section 3) that effectuates diffusion.

Because we focus on this mechanism, we exclude the possibility that firm 2 is rationed on the labour market by an additional assumption on firm investment behaviour, to which we turn now.

### Investment

After production has taken place, firms pay their workers and decide on investment. In order to simplify the analysis, we assume the extreme von-Neumann-hypothesis: Workers consume their entire income and capitalists do not consume.^5^ Further, firms only invest in their own business, an assumption that Silverberg (1984) terms ‘auto-catalytic self-reproduction’.^6^

For the investment process of firm *i*, its ‘individual’ expected rate of profit plays a decisive role, which depends (i) on the goods price it expects and the nominal wage rate it pays; (ii) on its technology; and (iii) on its capital utilisation rate:

4 $$ r^{e}_{i,t}= \frac{1-\frac{w_{i}}{p^{e}_{i,t}}l_{i}}{b_{i}}u_{i,t}, $$

where *u*_*i*, *t*_ ≤ 1 is firm *i*’s current capital utilisation rate, which is the ratio of actual output *x*_*i*, *t*_ to potential output *K*_*i*, *t*_/*b*_*i*_. Further, $p^{e}_{i, t}$ is the price firm *i* expected. Assuming that all firms have *static expectations*, it follows that $p^{e}_{i, t} = p_{t-1}$, where *p*_*t*−1_ is the last period’s uniform price. Clearly, a firm which is rationed on the labour market exhibits a rate of capital utilisation that is smaller than unity, or, stated differently an *actual* capital coefficient that is larger than it could be on purely cost-minimizing grounds. As a consequence, its ‘individual’ rate of profit is lower compared to the case in which it is not rationed. This detrimental effect of ‘new’ technology on ‘old’ capacity may be taken as a reflection of the process of creative destruction (Schumpeter 1934, 2010 [1942]); this is ultimately enforced by a lack of complementary inputs in the presence of a more profitable opportunity to employ them.

Further, firms take into account that the supply of labour limits the amount of capital which can be fully utilised. If firms are assumed to know the growth rate of labour supply, they can adjust to it by respecting the following inequality constraint:

5 $$ K_{i,t+1}\le\frac{\left( 1+n\right)N_{t}}{l_{i}}b_{i}, $$

where the right-hand side of the weak Eq. 5 gives the amount of capital needed to fully employ *all* workers available in period *t* + 1 using method *i*. For our case of just two rivals, this condition means a constraint on investment for the new firm 2 and implies that it is never constrained on the labour market in the way firm 1 is. For the old firm 1, constraint (5) is never binding because of Eq. 3a.

It follows that investment levels are determined by

6a $$\begin{array}{@{}rcl@{}} I_{1,t} &=& r^{e}_{1,t}K_{1,t} , \end{array} $$

6b $$\begin{array}{@{}rcl@{}} I_{2,t} &=& \min \left\{r^{e}_{2,t}K_{2,t}; \frac{\left( 1+n\right)N_{t}}{l_{2}}b_{2}-K_{2,t}\right\}, \end{array} $$

where *I*_*i*, *t*_ = *K*_*i*, *t* + 1_−*K*_*i*, *t*_ denotes *real* investment of firm *i*. Because the two firms differ in their technical conditions of production, they also differ in their ‘individual’ profit rates. And via Eq. 3a this difference translates into differential capacity accumulation of the two rivals. This is the basis for the well-known mechanism of differential growth (see Section 3).

The investment functions show that a lack of complementary inputs slackens capital accumulation in two different ways: If old firm 1 is rationed on the labour market, its lower rate of capital utilisation depresses its individual profit rate; this ‘de-valuation’ of its capacity results in decelerated accumulation. New firm 2 would face this problem only after it has become ‘too big’ and if it grows too rapidly. Because firms are treated as knowing the growth rate of labour supply but not the accumulation plans of rivals, the new firm avoids being rationed by adjusting its investments according to condition (5).

What has become visible so far is that the problem of a lack of complementary inputs may affect different types of firms in different ways. And that while some implications of an input bottleneck may instantly show economic effects, others remain in the shadow and enforce economic movements only after the system has passed some turning point. The third element of the model is goods market interaction, which is explained next.

### Goods market

In the goods market firms sell the part of their production which they do not use to grow capacity. It is assumed that all firms sell at the same price which is determined by *market clearing*.

At the time of market interaction total *real* supply *S*_*t*_ and total *nominal* demand *D*_*t*_ are given magnitudes determined by prior decisions on production and investment: The amount of goods supplied to the market equals total output minus total investments; and total *nominal* demand is the total wage bill since workers do not save and capitalists do not consume. Market coordination thus can only be brought about by a variation of the price.

For the goods market to clear, the price *p*_*t*_ adjusts such that real supply *S*_*t*_ and real demand *D*_*t*_/*p*_*t*_ coincide:

7 $$ p_{t}=\frac{D_{t}}{S_{t}}=\frac{w_{1}L_{1,t}+w_{2}L_{2,t}}{x_{1,t}+x_{2,t}-I_{1,t}-I_{2,t}}. $$

**Fig. 1:**
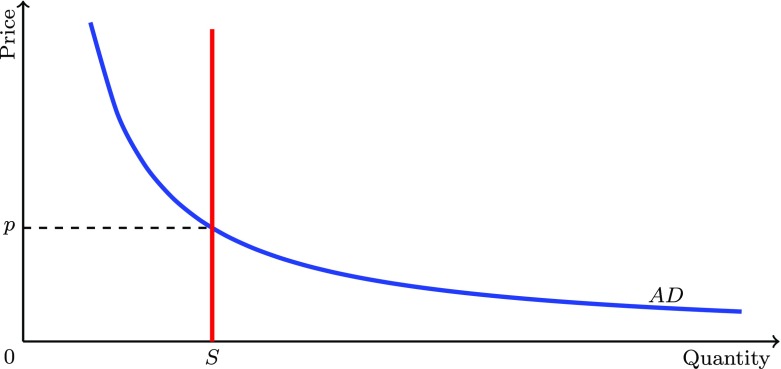
Aggregate real demand curve *AD*, aggregate real supply *S* and the market clearing price *p*

Figure 1 illustrates this: As the amount of goods supplied is fixed, the supply curve (the line *S*) is a vertical line. The aggregate real demand curve (the line *AD*) shows the relation between the amount of goods workers are able to purchase and the market price for their *given* nominal income. If the price rises, the quantity of goods a worker can buy falls. The price at which the two lines intersect clears the market and is the one at which all supplied goods change hands. Note that because the *Law of One Price* holds on the goods market but not on the labour market, employees of firm 2 receive a slightly higher real wage than employees of firm 1 such that there is inequality within the group of otherwise homogeneous workers.

One implication of this pricing rule greatly simplifies our analysis of the evolutionary traverse (see Section 3): As long as both firms’ investments are purely profit-led, the price does not change. This can easily be verified by applying Eq. 4 together with $I_{i,t}=r^{e}_{i, t}K_{i,t}$ for *i* ∈ {1, 2} to the pricing rule (7). As long as the price does not change, the growth rate of the new firm is constant and the rate at which the old firm accumulates changes due to rationing only. The stylized ‘mechanics’ at hand may thus put quantity adjustments unfolding in the course of adaptation into sharp relief.^7^

But this is not to say that price dynamics are not important here. To the contrary, as will be shown below, condition (5) sooner or later gets binding and the resulting price movements play a decisive role in restoring equilibrium. The fact that the price does not gradually adapt reflects one central theme of this study, namely that the forces which move the system may not remain the same over time. Rather, one force may shape economic movements in one phase, but at the same time it may pave the way for new forces. And if they gain momentum and prevail, the behaviour of the system may change and new phenomena may arise. Thus something can be learned from the study of the sequence of mechanisms and their interplay.

### Summary

**Fig. 2:**
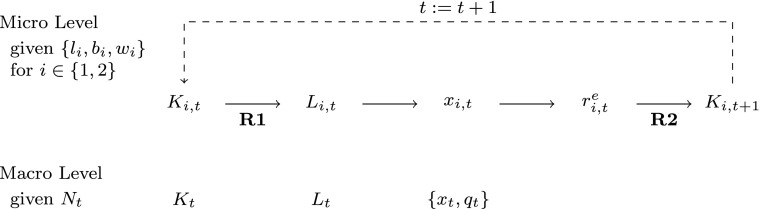
Logic of the model

Before we deal with this question, let us summarise the model in order not to lose sight of its data, variables and (behavioural) relations. Figure 2 illustrates the basic structure of the model. The givens consist of the set of firm-specific data {*l*_*i*_,*b*_*i*_,*w*_*i*_} where *i* ∈ {1, 2} and the rate at which the labour supply grows, *n*. The endogenous variables are the aggregate stock of machines $K_{t}=\sum \nolimits _{i}K_{i,t}$, aggregate employment $L_{t}=\sum \nolimits _{i}L_{i,t}$, aggregate output $x_{t}=\sum \nolimits _{i}x_{i,t}$ and the market share of firm 2, denoted by *q*_*t*_ = *x*_2,*t*_/*x*_*t*_, which shows the economic weight of the innovation.

Firm variables {*K*_*i*, *t*_,*L*_*i*, *t*_,*x*_*i*, *t*_}, where *i* ∈ 1, 2} from which aggregates are built, evolve according to two *micro rules*, which establish relations between firm variables: the method of production (1) and the investment functions (6a). The *macro rule* (2) determines labour supply, describes the environment in which firms act and defines channels through which they interact. There are two *interaction rules*: Eq. 3a establishes a functional relationship between firm variables yielding firms’ employment levels as outcomes of labour market interaction; and through coordination rule (7) firms interact via the market-clearing output price.

As already mentioned, for the case that labour supply grows at an exogenously given rate, two forms of rationing can occur, which are referred to as **R1** and **R2**:

**R1**: Firm 1 is rationed, if total labour demand is larger than supply. This implies that its capacity-determined employment level is strictly larger than its labour-supply-determined level ($L^{d}_{1,t}>N_{t}-L_{2,t}$).
**R2**: From condition (5) it follows that firm 2 is ‘investment rationed’ if its profit-determined level of investments is larger than its labour supply-determined full-utilisation level of investments.

## Adaptation and growth

This section turns to the dynamic process of adjustment in order to clarify the central forces and features of the evolutionary traverse. To put them into precise terms, we perform a ‘causal analysis’. For this purpose we compare the path of the adapting economy with its reference path. The adaptation path starts when some particular innovation disrupts the ‘old’ steady state and ends when a ‘new’ steady state is reached. Along the reference path no innovation takes place such that the economy remains in its ‘old’ steady state. The causal effect of the innovation then is the difference between the two paths.^8^ We assume that in the ‘old’ steady state all firms use the same method, namely ‘old’ method 1 with *l*_1_ and *b*_1_ as unit requirements and pay the same uniform and constant real wage rate *w*_1_/*p*; the price of the good equals unity in the old steady state. Further, there is full employment and the system grows at rate *g*_1_ = *r*_1_ = *n*. Consistency requires that *n* ≤ 1/*b*_1_.

Unlike most evolutionary selection models which start with given variety of methods, this approach requires us to not only consider the diffusion process, or the process of variety destruction, but also the process by which variety is created. For the study here, two questions of the innovation process are important. The first question concerns the conditions that the new method must satisfy in order to trigger a successful traverse (see Section 3.1). The second one pertains to the way in which the new method could emerge within a system where there are no unemployed resources and the effects this produces (see Section 3.2).

### Types of new methods

We distinguish between different types of new methods based on both their innovation bias and their economic superiority or inferiority.

Potential new methods, i.e. those not (yet) used, are grouped according to their *innovation bias*. The capital bias and the labour bias of the (new) method 2 compared to the (old) method 1 are given by the measures

$${\Theta}_{b}=\frac{b_{2}-b_{1}}{b_{1}}>-1\quad\text{and}\quad {\Theta}_{l}=\frac{l_{2}-l_{1}}{l_{1}}>-1. $$

where Θ_*b*_ (Θ_*l*_) is the relative gain in physical efficiency with respect to capital (labour). Note that both measures are assumed to be strictly larger than −1 so that both inputs are necessary to operate the new method.^9^ The bias of an innovation is defined by the combination of signs of the two measures. For example, new methods for which Θ_*b*_ = 0 and Θ_*l*_ < 0 are pure labour saving innovations. Table 1 lists all types innovation that are possible in a one-good model given the above condition. Below we show how the features of the innovation in terms of physical efficiencies, not just absolutely but relative to what is already there, crucially shape the path of diffusion and aggregate growth of the traversing economy.

**Table 1:** Causal effects in the innovation period.

Innovation type	Bias	R1	Δ_**x**, **0**_/**x**_**R**_	Δ_**L**, **0**_/**L**_**R**_
Capital saving and labour using	Θ_*b*_ < 0 , Θ_*l*_ > 0	Yes	–	0
Labour saving and capital using	Θ_*b*_ > 0 , Θ_*l*_ < 0	No	–	–
Pure capital saving	Θ_*b*_ < 0 , Θ_*l*_ = 0	Yes	0	0
Pure labour saving	Θ_*b*_ = 0 , Θ_*l*_ < 0	No	+	–
Combined factor saving	Θ_*b*_ < 0 , Θ_*l*_ < 0			
Neutral	Θ_*b*_ = Θ_*l*_ < 0	No	+	0
Dominantly capital saving	Θ_*b*_ < Θ_*l*_ < 0	Yes	+	0
Dominantly labour saving	Θ_*l*_ < Θ_*b*_ < 0	No	+	–

Second, we distinguish between methods which are economically superior to the old method and methods which are inferior. In contrast to an inferior method, a superior method is defined as one that would successfully spread and displace the old method along the traverse, if it were implemented. In our model a method is superior if it yields a rate of profit which is strictly higher than that of the old method and yet pays a wage rate that is sufficiently above the wage rate of the old firm (see Eqs. 3a and 6a).^10^ This condition implies that some (new) method 2 is superior to the (old) method 1 if

8 $$ r_{1}b_{1}\left( \frac{b_{2}-b_{1}}{b_{1}}\right)+\frac{w_{1}}{p}l_{1}\left( \frac{el_{2}-l_{1}}{l_{1}}\right)<0. $$

We see that the new method yields a higher rate of profit only if at least one of its two unit requirements is strictly smaller compared to the old method. Although a greater physical efficiency with respect to one input is necessary for a new method to be superior, in some cases it is not sufficient: If either one of the two unit requirements is higher for the new method and/or if its labour costs per unit of output are higher because of the wage differential, the ruling income distribution in terms of the wage share and the profit share determine the superiority or inferiority of the new method.

Hence, economic and institutional conditions play an important role for the success of new methods (Kurz 2008). The institutional conditions that play a role here are those which determine the size of *e*. As already noted, the size of *e* depends on the perfection of the labour market in terms of information and mobility of workers (Metcalfe and Steedman 2013, p. 169-170).

In the remainder of this study only the case of a superior method is studied. Notice that we also exclude ‘dynamic re-switching’ where a new method is superior initially but becomes inferior because the income distribution changes in response to its diffusion.

### The implementation period

At the beginning of the implementation period, which is period 0, firm 2 using the new method 2 is set up. By assumption it grows out of existing resources and comes into being through the ‘mutation’ of some small fraction of the ‘old’ capital stock. This ‘mutation’ is taken to be a singular, exogenous event and to some extent violates our assumption that installed machines cannot be transferred as between firms. But, in the words of Schumpeter, the important point is that an innovation requires “a ‘withdrawal of goods’ from their previous uses” (Schumpeter 1934, p. 108) as there are no unemployed resources available in a steady state. This is crucial because this influences the aggregate quantity effects of implementing a new method.^11^

In order to determine the effects of implementing a new method on aggregate output and employment, total output *x*_0_ and employment *L*_0_ of the adapting economy are compared to the reference economy, where in both cases the same stock of capital *K*_0_ and the same amount of labour *N*_0_ are available. The difference between the two cases is that in the adapting economy some fraction *K*_2,0_ is used by the new firm. The instant employment and output effects are described below. The findings are summarised in Table 1 for different types of new methods.

The effects of implementation on aggregate output and employment depend first of all on whether firm 1 is rationed already in the innovation period (**R1** holds) or not. **R1** holds in the innovation period if

$$\frac{l_{2}}{b_{2}}> \frac{l_{1}}{b_{1}}\qquad\text{or}\qquad{\Theta}_{l}>{\Theta}_{b}. $$

We see that **R1** holds if the labour intensity is higher for the innovating firm than for the old firm.

The instant employment effect is defined as the relative deviation of the implementation period’s aggregate employment *L*_0_ from the reference level *L*_*R*_ = *N*_0_ and is given by:

9 $$\begin{array}{@{}rcl@{}} \frac{{\Delta}_{L,0}}{L_{R}}=\frac{L_{0}-N_{0}}{N_{0}}= \left\{ \begin{array}{ll} 0 & \text{if  \textbf{R1} holds} \left( {\Theta}_{l}>{\Theta}_{b}\right),\\ \frac{K_{2,0}}{K_{0}}\left( \frac{{\Theta}_{l}-{\Theta}_{b}}{1+{\Theta}_{b}}\right) & \text{if  \textbf{R1} does not hold} \left( {\Theta}_{l}\le{\Theta}_{b}\right). \end{array} \right. \end{array} $$

Because a full employment reference path is assumed, the employment effect is either zero or negative. It is zero in cases in which the innovation is more labour intensive than the old one. Hence, if **R1** holds, full employment prevails in the implementation period, but some of firm 1’s machines lie idle. If a neutral innovation (Θ_*l*_ = Θ_*b*_) gets implemented, the employment effect is zero, but the capital stock remains fully employed in this period. If the innovation is less labour intensive (Θ_*l*_ < Θ_*b*_), installed capacity is fully utilised but some workers are unemployed.

We also see from Eq. 9 that the extent of what may be considered technological unemployment does not only depend on the innovation bias but also on firm 2’s initial capital share. For example, if the innovation is capital using and labour saving with Θ_*b*_ = 0.25 and Θ_*l*_ = −0.25, the employment effect is Δ_*L*, 0_/*L*_*R*_ = −0.1 for *K*_2,0_/*K*_0_ = 0.25. But if the whole capital stock mutates instanteneously (*K*_2,0_/*K*_0_ = 1), the employment effect is Δ_*L*, 0_/*L*_*R*_ = −0.4.

The instant output effect is the relative deviation of the implementation period’s aggregate output *x*_0_ from its reference *x*_*R*_:

10 $$ \frac{{\Delta}_{x,0}}{x_{R}}=\frac{x_{0}-x_{R}}{x_{R}}=\left\{ \begin{array}{ll} -\frac{K_{2,0}}{K_{0}}\left( \frac{{\Theta}_{l}}{1+{\Theta}_{b}}\right) & \text{if \textbf{R1} holds } \left( {\Theta}_{l}>{\Theta}_{b}\right), \\ -\frac{K_{2,0}}{K_{0}}\left( \frac{{\Theta}_{b}}{1+{\Theta}_{b}}\right) & \text{if \textbf{R1} does not hold } \left( {\Theta}_{l}\le{\Theta}_{b}\right). \end{array} \right.  $$

As we can see, the direction of the instant causal output effect is determined by the two dimensions of the innovation bias and how these compare with each other: If the innovation is more labour intensive than the old one, we know that **R1** holds. It follows from Eq. 10 that the labour bias Θ_*l*_ determines the direction of the output effect in this case. But if the innovation is less labour intensive and hence causes unemployment (**R1** does not hold), it is not the labour bias Θ_*l*_ but the capital bias Θ_*b*_ that determines the direction of the effect. It follows that in both cases the causal effect can be positive (or negative), although for different reasons (see Table 1).

The next section shows in what way economic circumstances, in particular whether there is full employment or not, interact with the process of differential growth that is initiated by the implementation of the new method and shapes its path of diffusion.

### The diffusion period

In this section we study the diffusion period during which the new method gradually gains weight in terms of its output share. This quantitative restructuring drives aggregate output and employment growth. The exercise below shows that the type of innovation under consideration crucially shapes the way its diffusion is effectuated and the effects this entails. As shown above, the implementation of some types of new methods makes workers redundant. It will turn out that the *instant* employment effect shapes the adjustment path.

The argument is the following: In case the new method is less labour intensive than the old method, i.e. if Θ_*l*_ < Θ_*b*_, implementation causes technological unemployment. This means that there is a phase during which no firm is rationed. As long as this situation prevails, diffusion is effectuated by differential capacity accumulation. Through this the new firm gradually gains economic weight in terms of its output share. This adjustment mechanism shapes things in the *re-absorption phase* where neither **R1** nor **R2** holds (see Section 3.3.3).

The re-absorption phase through which the system moves only if Θ_*l*_ < Θ_*b*_ eventually ends because the new firm grows at an above-normal rate and by doing so re-establishes full employment. The system then enters a different phase, during which firm 1 is rationed on the labour market, while firm 2 still expands at an above-normal rate by attracting workers from firm 1. This kind of predatory interaction shapes things during the *predation phase* where **R1** holds but **R2** does not (see Section 3.3.2).

Also this phase ends, because the new firm eventually exhausts its potential to grow at an above-normal rate by luring away workers from firm 1. If then condition (5) becomes binding and not only **R1** but also **R2** holds, the system enters the *restoration phase*, in which the innovator adapts his accumulation speed to the growth rate of labour supply. This leads to a strong fall in the output price as a consequence of which the system enters a new steady state (see Section 3.3.1).

#### The re-absorption phase

During this phase technological unemployment prevails because an innovation which is less labour intensive than the old method has been implemented. We call it the re-absorption phase, because jobless workers are gradually re-employed.

The reason why this happens is that the innovation yields an above-normal rate of profit because the market price does not change (see Section 2.3). As no firm is rationed their output growth rates are given by

$$g_{1} =\frac{x_{1,t+1}-x_{1,t}}{x_{1,t}}= r_{1} = n \qquad\text{and}\qquad g_{2}=\frac{x_{2,t+1}-x_{2,t}}{x_{2,t}}=r_{2}>n. $$

The growth rate of aggregate output is the weighted average of firm growth rates, where the weights are the output shares of firms. It can be expressed as

11 $$ g_{t} =\frac{x_{t+1}-x_{t}}{x_{t}}= n + \underbrace{ q_{t}\left( g_{2}-n\right)}_{\text{(re-absorption effect)}} > n, $$

where *q*_*t*_ is the market share of firm 2. We see that the aggregate growth rate is larger than *n*, which is the rate at which the reference economy grows, because of a positive ‘re-absorption effect’. The same holds for employment growth, which is the weighted average of firm growth rates with firms’ employment shares as weights. Isolating the re-absorption effect gives

$$g_{L,t} =\frac{L_{t+1}-L_{t}}{L_{t}}= n + \underbrace{ q_{t} \frac{1+{\Theta}_{l}}{1+q_{t}{\Theta}_{l}}\left( g_{2}-n\right)}_{\text{(re-absorption effect)}} > n, $$

and shows that its extent also depends on the innovation’s labour bias Θ_*l*_: The more the innovation saves on labour, the slower job growth is. This indicates that the length of the re-absorption phase depends on the type of innovation.

The structure of production in terms of output shares of the two rivals changes due to the mechanism of differential accumulation at a rate which is proportional to the difference in profit rates:

12 $$ \frac{q_{t+1}-q_{t}}{q_{t}}=\frac{g_{2}-g_{t}}{1+g_{t}}= \left( 1-q_{t}\right)\frac{ \left( r_{2}-r_{1}\right)}{1+g_{t}}. $$

Equation 12 defines the diffusion path and shows that the new method displaces the old along a sigmoid curve. This logicstic replacement pattern is a typical result of evolutionary models of the variation cum selection kind for the case of two rival methods (Metcalfe 2008). However note that it is not a simple ‘S’-shaped logistic curve, because the aggregate growth rate *g*_*t*_ is not constant but increases over time (Metcalfe and Steedman 2013, p. 164).

Summing up, the re-absorption phase appears bright and prosperous: The economy grows at a rate which is always above of what was feasible before the innovation occurred. But note that this is only possible if the emergence of the innovation at first caused unemployment. Further keep in mind that the mechanism of differential growth shapes economic movements and causes a logistic pattern of diffusion.

#### The predation phase

As differential growth eventually restores full employment, the system enters the predation phase, which is defined by the fact that **R1** holds but **R2** does not. This implies that a new force sets in and shapes the course of things.

As now full employment prevails, the causal employment effect is zero. We thus focus on the causal output effect. The explanation of the causal output effect begins with the remark that also in the predation phase the market price does not change. Hence firm 2’s rate of profit remains constant and above *n*. Yet, firm 1’s position is less favourable as it is no longer able to maintain full capital utilisation. Idle capital in turn forces down its individual rate of profit such that

$$r_{1,t}<n \qquad\text{and}\qquad r_{2}>n.$$

This detrimental effect of ‘new’ technology on ‘old’ capital ensures that the speed at which firm 1 accumulates capacity abates.

But this is not all that happens. Predatory interaction on the labour market by Eq. 6a implies that the growth rate of the old firm now depends on the growth rate of the new firm:

13 $$ g_{1,t}= n + \underbrace{\left( -1\right) \frac{q_{t}}{1-q_{t}}\left( 1+{\Theta}_{l}\right)\left( g_{2}-n\right) }_{\text{(predation effect)}}<n. $$

Since *g*_2_ > *n*, this equation shows that the faster the innovator grows the slower the old firm expands in terms of output. By predation of workers, firm 2 pushes down firm 1’s rate of output growth and thereby continues to be able to realize an ‘above-normal’ growth path. The mechanism of ‘growth predation’ thus leads to a situation in which

$$n>g_{1,t}\qquad\text{and}\qquad g_{2}>n, $$

where *g*_2_ = *r*_2_.

This direct and one-sided dependency shown in Eq. 13 is a novel feature in the context of variation-cum-selection models. What difference does it make for adaptive growth and the path of diffusion?

For the system as a whole, growth predation affects aggregate output growth by

14 $$ g_{t} = n + \underbrace{\left( -1\right)q_{t}{\Theta}_{l}\left( g_{2}-n\right)}_{\text{(predation effect)}}. $$

We see that adaptive growth may differ from reference growth *n* due to the ‘predation effect’ of Eq. 14. Since *g*_2_ > *n*, the sign of this effect is determined by the innovation’s labour bias Θ_*l*_ only. Hence innovations do not necessarily entail an expansionary tendency in the predation phase: Only if the innovation saves on labour (Θ_*l*_ < 0) the economy experiences ‘above-normal’ growth; but if a labour-using innovation (Θ_*l*_ > 0) gains economic weight, ‘below-normal’ growth results; and if Θ_*l*_ = 0 the economy grows at its normal rate *n*. Therefore there are cases in which the aggregate output growth rate flips from an above-normal to a below-normal level, since re-absorption growth is always above the normal level.

The reason why the labour bias of the innovation is a major determinant of aggregate growth is that the economy hits the full-employment ceiling as it passes from the re-absorption to the predation phase. This means that total output is given by $x_{t}=N_{t}/\bar {l}_{t}$, where $\bar {l}_{t}$ is the average labour coefficient, defined by $\bar {l}_{t}=\left (1-q_{t}\right )l_{1}+q_{t}l_{2}$. Taking the growth rate of the average labour coefficient reveals that output growth rate is smaller than *n* if a labour-using innovation gains economic weight.

The fact that the system hits the full-employment ceiling does not only change the determinants of adaptive growth but also the adaptation mechanism. Because growth predation breaks the one-to-one relation between the profit differential and the growth differential, it undermines the ‘pure logistic law’ as a driver of restructuring. To see this, let us turn to the evolution of employment shares, which are more informative than the corresponding output shares here: Let employment share of firm 2 be *q*_*L*, *t*_ = *L*_2,*t*_/*L*_*t*_ and let Λ_*t*_ denote the rate at which it changes.^12^ The rate of change in employment shares for the re-absorption phase and the predation phase then are:

15 $$ {\Lambda}_{t}= \frac{q_{L,t+1}-q_{L,t}}{q_{L,t}}=\left\{ \begin{array}{ll} \left( 1-q_{L,t}\right)\frac{\left( r_{2}-r_{1}\right)}{1+g_{L,t}} & \text{in the \textbf{re-absorption} phase}, \\ \frac{g_{2}-n}{1+n} & \text{in the \textbf{predation} phase}, \end{array} \right.  $$

where *g*_*L*, *t*_ is the rate at which total employment grows in the re-absorption phase (*L*_*t*_ < *N*_*t*_). From Eq. 15 it follows that the same logistic process effectuated by differential growth as in Eq. 12 shapes employment shares in the re-absorption phase. In contrast, in the predation phase the problem of labour shortage offsets this mechanism and causes a different adaptation pattern. Economic movements now result from growth predation, a mechanism which shows an exponential pattern of restructuring, where the rate of change is constant.^13^

One may infer from this finding that if bottlenecks and predatory interaction on input markets play a role, the pure logistic law of replacement may not always hold if looked upon from a purely theoretical perspective. For example, in a world in which industries are interconnected, imbalances of supply and demand of complementary inputs may shift the probability in favour of exponential replacement patterns rather than logistic ones.

#### The restoration phase

So far we have treated the case in which the old firm is not rationed and the case in which it is rationed and showed how the re-absorption phase paves the way for the predation phase. This section now turns to the case in which not only firm 1 but also firm 2 is affected by the labour inflexibility assumption. Hence both **R1** and **R2** hold.

In Section 2 we argued that the new firm is able to avoid being rationed in the way the old firm is. This assumption resides in constraint (5), which enters firm 2’s investment function (6b). Then, **R2** holds if firm 2’s profit-determined level of investments is larger than its labour supply-determined full-utilisation level. In the first period where **R2** holds, say *T*, firm 2’s real investment therefore is given by

$$I_{2,T}= \frac{\left( 1+n\right)N_{T}}{l_{2}}b_{2}-K_{2,T} < r^{e}_{2,T}K_{2,T}. $$

This implies that firm 2 is ‘investment rationed’ in the sense that the evolution of labour supply de-motivates the realisation of potential growth determined by the profit rate. Hence the innovating firm’s profit-led growth regime ends during the passage from the predation phase to the restoration phase.

That the system necessarily passes over from one to the other is due to the fact that the potential for firm 2 to grow at an above-normal rate by luring away workers from firm 1 eventually exhausts. That firm 2 is ‘investment rationed’ implies that its growth rate is

16 $$ g_{2,T}=\left( 1+n\right)\frac{\bar{l}_{T}}{q_{T}l_{2}}-1, $$

where *q*_*T*_ < 1. Because in period *T* + 1 firm 2 owns exactly that amount of capital required for employing all workers, output of firm 1 in period *T* + 1 is zero and *g*_1,*T*_ = −1. This implies that in period *T* + 1 the innovation is fully absorbed into the system. ‘Old’ capacity now is economically obsolete in the sense that ‘new’ capacity has grown big enough to employ the whole labour force.

What completes adaptation is the fact that in the restoration phase the output price erodes: If **R2** holds, $g_{2,T}<r^{e}_{2,T}$, which means that the amount of goods supplied is greater than the amount which would maintain a stable nominal price. Given the assumption of perfect coordination by Eq. 7, a price *p*_*T*_ is established which is smaller than the price which prevailed during the re-absorption and the predation phase. Because the price ‘jumps’ to a lower level, the real wage rate and the real costs of production increase. The distributional consequences of innovation now affect not only workers employed by the new firm (through the wage differential) but also workers still employed by the old firm, which therefore is at risk of losing its economic viability.

As noted above, output of firm 1 in period *T* + 1 is zero. Its capital stock is economically obsolete and hence ready to expire physically. If free disposal is assumed, the problem of getting rid of it will not have significant economic effects. Because firm 1 vanishes (*q*_*T* + 1_ = 1), it follows from Eq. 16 that *g*_2,*T* + 1_ = *n*. Also for this period **R2** holds which means that the price drops again; the price which gets established, say *p*_2_, re-establishes a new steady state path along which the profit rate equals *n* and the real wage rate is given by (*w*_1_ *e*)/*p*_2_ = (1−*n* *b*_2_)/*l*_2_.

We may conclude the discussion of the restoration phase by pointing out that one theme of this study, namely that adaptation forces may not remain constant but change conditions such that new forces set in and new phenomena arise, appears here in the form of non-steady price dynamics: The price is stable first, but strongly reacts after the system passed some turning point, which is reached due to the inner logic of change. Above all, this hints at the uneven nature of economic change we ought to explain.

## Conclusions

In this paper we clarified the role of a resource constraint for the evolutionary adjustment process triggered by the arrival of new methods of production. By means of a causal analysis we have obtained two main results.

First, the nature and effects of adaptation to a new method crucially depend on whether surplus labour exists or not. Concerning the nature of evolutionary adjustments we have shown that if there is surplus labour, differential accumulation leads to a logistic pattern of restructuring. But in the case of a labour shortage, growth predation through which firms’ output growth rates become interdependent, leads to an exponential replacement pattern. Through a comparison of re-absorption growth and predation growth the state of labour supply in relation to demand has also been shown to play an important role for the effects of new methods on aggregate growth along the traverse.

Second, different types of innovations lead to different adaptation paths and effects. Some innovations cause technological unemployment, which is eventually removed through the new firm’s above-normal ability to accumulate. Overall, adaptive growth is not steady and it is not necessarily the case that innovations boost aggregate growth.

By way of a conclusion, even in simple models like the one studied here the effects of innovations are hard to assess. From the objectivist position taken up here, this is so because diffusion is a time-consuming process such that effects extend over time; and because effects depend on the features of the innovation, not just absolutely but relative to what is already there, as well as on the economic circumstances into which they are born and spread. Hence, without taking economic circumstances into account we cannot expect to know how innovations will change the system. And even if the diffusion of an innovation causes some pattern to persist for some time, it may prove a bad guide for the future because forces behind patterns may revise the economic circumstances on which they rely.

## Appendix: Full Automation

Here we deal with the case of a fully automated method and show that the dynamic process of adjustment to it differs fundamentally from the cases discussed so far. The (hypothetical) case where machines have completely replaced human labour is discussed by Ricardo (1951, Works VIII, pp. 399–400). He perceived full automation as the ultimate result of mechanisation that took place in his times and pointed out that this would have tremendous implications for the distribution of income (Kurz 2015b, p. 823).

The fully automated method, or ‘robot method’, produces without labour, that is by means of *unaided capital* (*l*_2_ = 0 and *b*_2_ > 0 ). We may think of ‘robot capital’ as autonomously acting computer-controlled equipment, which is able to self-replicate.

As above, we assume that it is able to pervade the system (hence *r*_2_ = 1/*b*_2_ > *n*) and that the initial stock of robots is constructed by means of ‘old’ capital. According to Eq. 9, this causes technological unemployment. But in contrast to the cases above, unemployment is not transitory but is persistent because the new robot firm does not create jobs. Full employment could be restored if the old firm increases its rate of job creation, which is only possible through a (temporary) real wage cut and hence entails a distributional conflict, namely between employed workers, who want to maintain their wage rate, and unemployed workers, who want jobs to be created.

Assume that our investment hypothesis adopted so far, namely that *g*_2_ = *r*_2_, applies to the new fully automated firm. This means that its investments equal its output because it pays no wages. As it neither demands labour nor offers goods to consumers it is completely disconnected from the rest of the system (see Metcalfe and Steedman 2013, p. 175) such that the old firm continues to grow at rate *n* while the new firm grows at a rate larger than *n*. But, as there is no competition for labour, the economy never enters the predation and restoration phase, which implies that the gradual diffusion of the robot method (i) does not drive the old method from the market in absolute terms; (ii) does not raise the real wages, but (iii) *permanently* increases the average profit rate and the profit share, which approaches unity in the limit. This is in sharp contrast to the cases discussed so far, where workers eventually gain through the price fall and where the (average) profit rate returns to the normal level eventually (see Section 3.3.3).

Assume that *n* < *g*_2_ < *r*_2_ and that the fully automated firm invests a constant fraction of its output *x*_2_ (which is equal to its profits) into its own business: *g*_2_ = (1−*c*)*r*_2_ = (1−*c*)/*b*_2_, where 0<*c* ≤ 1 is the fraction of output not invested; in period *t* this amount is *c* *x*_2,*t*_. If robot owners consume it directly, we would have a traverse similar to the case where *g*_2_ = *r*_2_, although with a lower diffusion speed. To the contrary, if *c* *x*_2,*t*_ is put on the market the situation is different: The market clearing price falls, because this increment of supply of goods is not accompanied by additional aggregate demand (which is total nominal income of workers). The price drop increases the real wage rate of the old firm and decreases its rate of profit accordingly. As a consequence, the below-normal rate at which it creates new jobs leads to mass unemployment. Hence, as long as the price is not zero the system experiences a rather ‘dystopian’ traverse with high and persistently increasing unemployment; at the same time workers who still have a job gain from the drop in the price.

The situation in which the robot firm sells part of its output to workers will be better in terms of employment, if robot owners spend their earnings (partly) on something that increases the volume of paid labour. For example, robot owners could demand a personal service which can be produced by means of unassisted labour, i.e. Ricardo’s ‘menial servants’ (Ricardo 1951, Works I, p. 392). In our model, this would mean that the volume of paid work increases through the invention of a new use of labour. The transition towards a situation where the production of the consumed good is fully automated then involves the rise of a service industry, which fulfils desires of robot owners. Whether the demand for servants increases at the normal level or not, is not so clear. Further, total demand for service labour may be bounded from above, if consumption takes time (Steedman 2001) and robot owners eventually run out of time to enjoy all the services they could afford. If the time constraint becomes binding, the number of servants employed stagnates unless new and more labour-using services are invented.

We may conclude our crude discussion of full automation by pointing out a possible use for goods produced by means of robots through which workers gain from automation and eventually have no longer the need to work. A portion of output *c* *x*_2,*t*_ of the robot firm may be taken away, either by taxing robot owners or by their ‘voluntary charity’, and may be distributed equally across the population. Such a ‘basic income’ per head *B* *I*_*t*_ = (*c* *x*_2,*t*_)/*N*_*t*_ will be tiny (and, perhaps, too low to make for a living) in the beginning since robotised production is small. But if *g*_2_ > *n* still holds, however, basic income per head steadily (and exponentially) increases. Eventually, the basic income rises to a level which exceeds the maximum amount of goods a worker can consume given his time constraint. Then the old firm can be shut down since going to work is no longer necessary.

## Footnotes

1 The first evolutionary diffusion model dates back to Nelson (1968). Further important contributions include Nelson and Winter (1982, chap. 10), Soete and Turner (1984), Silverberg (1984), Englmann (1992), Metcalfe (1997, 1998), Nelson and Pack (1999), Metcalfe and Steedman (2013); see also Haas (2015). See Metcalfe (1998) for a concise elaboration of the economic theory of selection. For an overview of the evolutionary perspective on growth and technical change see Santangelo (2003) and Silverberg and Verspagen (2005).

2 Evidence suggests that the case of strong ‘incumbent inertia’ is not purely hypothetical; see for example Gilbert (2005) and the references he gives.

3 Note that the assumption of an exogenous population growth rate is quite at odds with the classical perspective, in which the workforce endogenously adjusts to the pace of capital accumulation (Kurz 2008, 2010). It may be motivated by the idea that the structure of productive activities changes faster than the factors which determine population growth.

4 Note that here the explanation of the wage differential has nothing to do with the skills of workers or the quality of jobs; see Kurz and Salvadori (1995, chap. 11) for an analysis of persistent forces which regulate the structure of relative wages in the long run.

5 The classical saving hypothesis according to which workers do not save and investments depend on capital incomes is a typical assumption made in evolutionary growth models; see e.g. those cited in footnote 1 and Dosi et al. (2010).

6 See Soete and Turner (1984) for a discussion of investment flow adjustments in the context of diffusion and selection.

7 This feature of the model mimics the hypothesis of Metcalfe and Steedman (2013) who treat the real wage rate, except for a small wage premium, as constant during the adjustment process on the basis of the principle of the marginal firm.

8 This method has been employed by Kalmbach and Kurz (1992) in their empirical study of employment effects of diffusion of new methods of production. Hicks (1983) describes the *causal analysis* as follows: We compare two alternative paths that extend into the future. Along one of those paths some new ‘cause’ is not operating; along the other it is. The difference between the paths is the effect of that cause. The difference itself extends over time [and] it is the *whole* of the difference between the paths which is the effect of the cause (p. 109; *Hicks’s italics*).

9 The appendix on page 19 discusses the case of a fully automated method (Θ_*l*_ = −1) and shows that it differs fundamentally from cases where the new method requires labour.

10 In the following, *e* = *w*_2_/*w*_1_ > 1 denotes the ratio of the two wage rates and reflects the assumption that the new firm pays a higher wage rate in order to compete away workers from the old firm; the size of *e* is assumed to be constant and given.

11 The argument that broader economic conditions play an important role for the process of innovation and effects has been emphasised by both Schumpeter (1934, 1939) and Spiethoff (1925). Whereas Schumpeter assumed that innovations are introduced into a situation of full employment, i.e. what he termed a ‘circular flow’, Spiethoff argued on empirical grounds that innovations normally are born into a world in which some economic resources lie idle. This difference led them to put forth different views on innovation-driven change (Kurz 2015a).

12 The employment share relates to the output share by $q_{L,t} \bar {l}_{t}= q_{t} l_{2} $.

13 Note that in terms of capital shares *K*_2,*t*_/*K*_*t*_ and *K*_1,*t*_/*K*_*t*_, the logistic law remains intact, although the adaptation speed is higher than in the re-absorption phase. But the point is that the change in capital shares no longer drives structural change.
